# Synthesis of Honeycomb-Like Carbon Foam from Larch Sawdust as Efficient Absorbents for Oil Spills Cleanup and Recovery

**DOI:** 10.3390/ma11071106

**Published:** 2018-06-28

**Authors:** Jia Tan, Wei Li, Chunhui Ma, Qiong Wu, Zhou Xu, Shouxin Liu

**Affiliations:** College of Materials Science and Engineering, Northeast Forestry University, Harbin 150040, China; tanjiacn@outlook.com (J.T.); liwei19820927@126.com (W.L.); mchmchmchmch@163.com (C.M.); wuqiong0506@hotmail.com (Q.W.); xuzhou0194@126.com (Z.X.)

**Keywords:** oil absorbents, carbon foams, hydrophobicity, oil cleanup, wood materials

## Abstract

Hydrophobic oil absorbents with interconnected porous structure have been widely used in dealing with the pervasive environmental issue of oil spills. In this work, hydrophobic foams with 3D interconnected porous honeycomb structures of liquefied-larch-based polymer foam (LLB-PF) and its carbonized product liquefied-larch-based carbon foam (LLB-CF) was prepared from larch sawdust waste and used for oil and organics separation. The results revealed that the 3D interconnected and open-cell honeycomb structure of LLB-PF was formed simultaneously during self-foaming, which remained intact even after carbonization. The two ultralight foams, especially LLB-PF, exhibited remarkable oil/water selectivity. The foams exhibited efficient and rapid absorption capacities, not only for oils but also for organic solvents. LLB-PF and LLB-CF could absorb tetrachloromethane and epoxidized soybean oil up to 88 and 153 times their own weight, respectively. The recycle tests showed that LLB-PF and LLB-CF exhibited excellent absorption capacities even after five cycles, demonstrating an excellent cyclability. The high oil and organic solvent absorption performance along with the renewable and low-cost starting materials positions LLB-PF and LLB-CF foams as promising candidates with great potential for oil and organics cleanup.

## 1. Introduction

The development of industrialized societies was accompanied by oil and organic pollution, including frequent oil spills, leakage of organic solvents and oily industrial wastewater, causing severe environmental and ecological problems [1,2,3,4]. It has become a worldwide challenge to be addressed, which calls for the generation of novel techniques that can effectively recycle oil and organic solvents from aqueous media [5,6,7,8]. Various techniques, including oil skimmers, oil containment booms, dispersants, gravity processing, ultrasonic separation, membrane filtration, biological treatment, solidifiers and absorbents have been used to solve the problems of oil and organic solvent pollution [9,10,11,12,13,14,15,16,17]. Among them, physical absorption by efficient, affordable and reusable foam absorbents, which can effectively remove oil and organic solvents from the water phase, is attractive due to its simple process and the easy reclaiming of valuable resources [1,8,18,19].

Foam absorbents composed of interconnected open-cell structures are new-generation materials with outstanding properties that have been extensively investigated, offering potential applications as catalyst supports [20,21], supercapacitors [22,23,24], thermal energy storage [25,26], and absorbents [27,28,29]. In particular, their hydrophobicity, high absorptive capacity, low density, high porosity and excellent thermal stability make them attractive candidates for oil and organic solvent clean up. Furthermore, foam materials with an interconnected structure and hydrophobic surface have been successfully used to separate oil and organic solvents from water (Table 1). Under the cooperation of surface chemical compositions and micro-/nanostructure, foam absorbents with a hydrophobic property are only wetted by oil and organic solvents, providing efficient channels and spaces within the interconnected structure for the diffusion and enrichment of the organic phase [2]. However, the preparation of such foam absorbents requires non-renewable or expensive precursors, large amounts of chemicals and complex equipment, constraining their bulk production for practical applications. Therefore, it is highly desirable to explore an affordable and friendly strategy for bulk production of renewable foam absorbents.

Recently, the application of biomass for the preparation of advanced materials has attracted significant concerns [30,31,32]. For example, cotton [33], kapok [34] and lignin [35] have been used to prepare oil absorbents, but the use of those valuable biomass resources in the recovery of oil and organic solvents may be considered wasteful. Considering the large quantities available and the renewable characteristics of biomass such as waste wood, the transformation of waste wood to make advanced foam materials are considered promising [36]. However, it is hard to prepare foam absorbents with a regular morphology and a controlled porous structure directly from raw biomass materials [37]. According to our previous studies, biomass liquefaction technology laid the foundation for controllable morphology and structure, overcoming the intrinsic drawbacks of biomass as a raw material. Recently, in our group, carbon foams (CFs) with well-connected honeycomb cells were prepared from waste larch sawdust [38]. By addition of polyethylene glycol (PEG), the pore structure could be controlled at nanometer scale and the CFs obtained exhibited excellent adsorption performance for vitamin B12. The cell sizes in CFs were in the range 100–350 µm, which is much larger than in many other foam absorbents. These large cells offer potential to store large amounts of solvents, far higher than the adsorbent material weight. However, the closed-cell structure within this material and its intrinsic hydrophilicity encouraged us to explore a new method for developing hydrophobic foam absorbents with an integrated, open-cell structure.

Here, we develop two types of ultralight, hydrophobic and recyclable foam absorbents: liquefied-larch-based polymer foam (LLB-PF) and its carbonized product liquefied-larch-based carbon foam (LLB-CF), with 3D interconnected honeycomb open-cell structures, were prepared from waste larch sawdust. The absorptive capacities and recyclabilities of these foam absorbents were tested by absorption of various oils and organic solvents, followed by repeated desorption and absorption cycles. Their capability for use in separation was demonstrated by applying them as filters for the direct separation of a mixture of water and organics.

## 2. Materials and Methods

### 2.1. Materials

Larch sawdust (30–80 mesh) was provided by a local timber mill. Phenol (AR grade) and formaldehyde (AR grade) were purchased from Kermel Chemical Corporation (Tianjin, China). Tween 80 was purchased from TianLi Chemical Corporation (Tianjin, China). Coal oil and diesel oil were provided by the China National Petroleum Corporation (Harbin, China). Olive oil and soya bean oil were provided by Aladdin Industrial (Shanghai, China). Other chemicals were obtained from Kermel Chemical Corporation. All reagents were used without further purification.

### 2.2. Preparation of LLB-PF and LLB-CF

Liquefied larch sawdust was synthesized according to methods described in a previous report [38]. A total of 100.0 mL of formaldehyde solution (37 wt %), containing 1.0 g sodium hydroxide and 20.0 mL distilled water, were added to the liquefied larch sawdust and stirred to obtain a homogeneous mixture. The mixture was reacted at 55–60 °C for 2 h, and then heated under reflux at 95–98 °C for 1 h. The product was cooled to 60 °C, and the pH was adjusted to neutral using hydrochloric acid. Water was removed from the mixture by vacuum distillation, leaving behind a polymer with suitable viscosity. Next, 1.0 mL of Tween 80 was mixed with the polymer after cooling to 32 °C, and 40.0 mL of n-pentane was immediately mixed with the polymer and stirred vigorously to form a homogeneous mixture. After that, 8 mL of sulfuric acid (50 wt %) was added drop-wise to this polymer mixture, and then immediately poured into an open-topped square plastic box. The polymer mixture foamed and solidified upon heating at 60 °C for 24 h, denoted as LLB-PF. The polymer foam was then transferred to a tubular furnace for pyrolysis under a nitrogen flow and heated to 600, 700, 800, 900 and 1000 °C at 5 °C/min and held at these temperatures for 2 h, respectively. The resultant carbonized foams were denoted as LLB-CFx, where x indicates the last pyrolysis temperature. LLB-CF refers to LLB-CF900 when there was no special markup.

### 2.3. Characterization of LLB-PF and LLB-CF

A scanning electron microscope (SEM, Quanta 200, FEI, Amsterdam, The Netherlands) and transmission electron microscope (TEM, Tecnai G2 F20 S-TWIN, FEI, Hillsboro, OR, USA) were used to observe the morphologies of the prepared foams. A Fourier transform infrared spectroscope (FT-IR, Nicolette 6700, Nicolet, Madison, WI, USA) was used to evaluate the chemical structure of foams. A thermogravimetric analyzer (TGA 209 F3, Netzsch, Selb, Germany) was used to monitor the behavior of foams heating in nitrogen and air. Contact angles of foams were measured on a contact angle system (DSA25, Krüss, Hamburg, Germany) at room temperature, using 5-μL droplet volumes. The bulk densities of the foams were determined by weighing a block of known dimensions, according to the standard method from ASTMD1622-03 [39].

### 2.4. Absorption Tests

The absorption capacities (q) of LLB-PF and LLB-CF were measured for various types of oil and organic solvent. In a typical absorption test for LLB-CF, a given LLB-CF sample (mC0) was immersed in an oil or organic solvent for 1 min, then removed and quickly weighed (mC1). The q values for LLB-PF and LLB-CF were calculated according to the following equation (where qR and qC is the absorption ratios of LLB-PF and LLB-CF, respectively):

qR = (mR1 − mR0)/mR0 × 100%(1)

qC = (mC1 − mC0)/mC0 × 100%(2)

### 2.5. Recyclability Tests

The recyclability of LLB-PF and LLB-CF were evaluated by cyclic absorption–distillation or absorption–combustion measurements. In brief, n-heptane-filled foams were distilled to remove absorbed n-heptane, while ethanol-filled LLB-CF was directly combusted in air, and the clean samples then reused in the next cycle. The weight of sample was recorded before and after each cycle, the recyclable tests were carried out for five times.

### 2.6. Separation Tests

The separation abilities of LLB-PF and LLB-CF were demonstrated by direct separation of a mixture of water and organics stained with Sudan 3, which was poured into one side of the trough, then left to stand for a simple gravity-driven separation to completion. The separation device consisted of LLB-PF and LLB-CF plates, respectively. Both plates were of thickness 10 mm, and were bonded together with silicon sealant to form a trough.

## 3. Results and Discussion

### 3.1. Morphology

**Figure 1:**
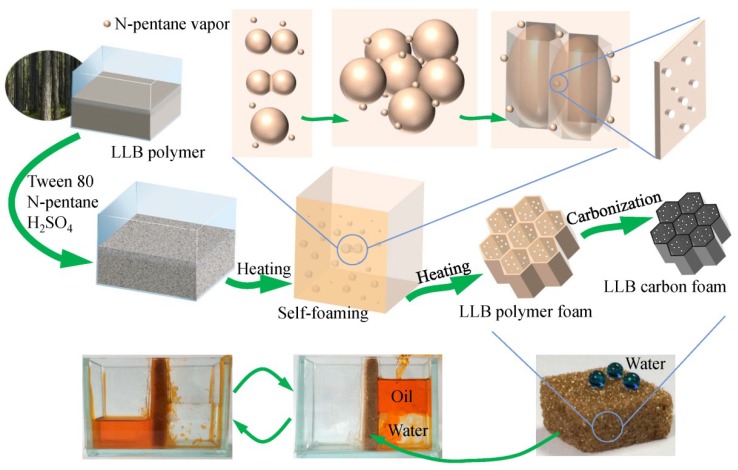
Fabrication process of liquefied-larch-based polymer foam and its homologous carbon foam.

The fabrication process of the liquefied-larch-based polymer foam (LLB-PF) and liquefied-larch-based carbon foam (LLB-CF) by using larch sawdust as the raw material is shown in Figure 1. The LLB polymer solution was prepared through the acid-catalyzed phenol-liquefaction and resinification of larch sawdust. The as-prepared liquefied larch was mainly composed of degraded lignin and degraded cellulose as well as other fragments with newly generated multiphenolic rings, which were more reactive toward formaldehyde and could help the resin polymer cure faster [40,41]. Emulsifier, foaming agent and curing agent were added into the LLB polymer, followed by vigorously stirring to form a homogeneous mixture. Afterward, the solution mixture was poured into an open-topped square plastic box, foamed and solidified upon heating at 60 °C for 24 h. Many tiny bubbles appeared with an increase in temperature of the polymer mixture. Larger bubbles then appeared as adjacent bubbles coalesced. With the resin polymer curing closed to complete, the viscosity of the polymer was growing, the bubbles fusion speed was getting slower, until the curing was completed. Large bubbles formed giant honeycomb cells hole, pores connecting adjacent cells were formed by tiny bubbles but not fully coalesced. The LLB polymer and bubbles can only inflated longitudinally in the influence of the square plastic box. After self-foaming and solidification, the 3D interconnected honeycomb open-cells and pore structures were formed simultaneously through cooperative volatilization of n-pentane and foam homogenization with Tween 80. After carbonization, the interconnected open-cells in LLB-CF remained intact, although the cells were smaller than in LLB-PF. LLB-CF inherited the honeycomb structure from LLB-PF and the foam morphology was not destroyed after heat treatment.

**Figure 2:**
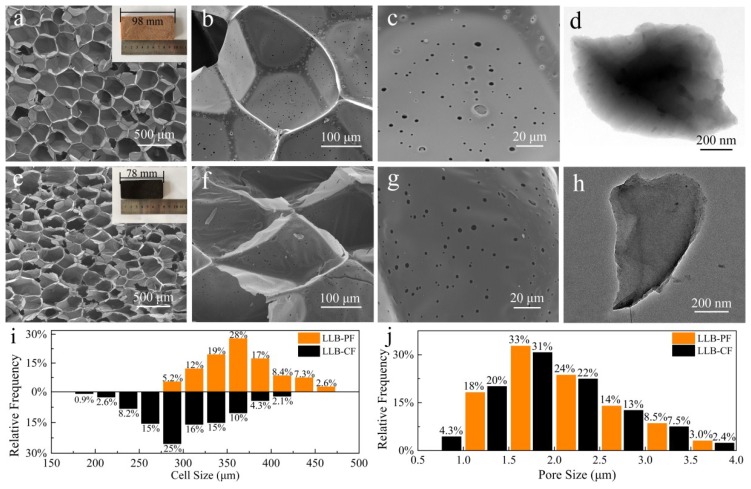
Morphologies and microstructure size distribution of LLB-PF and LLB-CF. (Inset (**a**,**e**)) Digital images of: LLB-PF (**a**); and LLB-CF (**e**); (**a**–**d**) SEM and TEM images of LLB-PF; (**e**–**h**) SEM and TEM images of LLB-CF; and (**i**,**j**) cell and pore size distribution of LLB-PF and LLB-CF.

To further understand the microstructure of as-prepared foams, SEM and TEM images and microstructure size are shown in Figure 2. It was clear that, after carbonization at 900 °C for 2 h, other than volume shrinkage of the LLB-PF caused by volatilization of unstable polymer components during carbonization, there were no apparent differences between the appearances and morphologies of the materials. The SEM images showed that both LLB-PF and LLB-CF exhibited 3D interconnected honeycomb open-cell structures. At a higher magnification (Figure 2c,d,g,h), the cell walls of both foams were very smooth, and there were abundant circular holes so called pores randomly distributed on the cell walls, connecting adjacent cells and making the inner cell accessible to the solution. Owing to the self-foaming process, the as-prepared foams exhibit low density and porous structure (Figure 2i,j). LLB-CF had a lower density of ~9.7 mg/cm^3^, measured using block volumes and weights, while the block density of LLB-PF was ~13.5 mg/cm^3^. The cell sizes in LLB-PF mainly ranged 300–450 µm, with pore sizes ranging 1.0–3.5 µm. The cell sizes in LLB-CF mainly ranged 225–375 µm, with pore sizes ranging 1.0–3.5 µm.

### 3.2. Surface Wettability and Functional Groups

**Figure 3:**
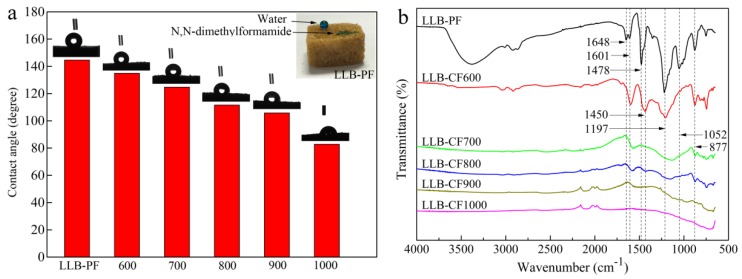
Surface wettability and FT-IR spectra of LLB-PF and LLB-CF at different pyrolysis temperature: (**a**) water contact angle measurements of LLB-PF and LLB-CF, the inset is a photographic image of a water droplet supported on LLB-F, and a drop of N,N-dimethylformamide absorbed by LLB-PF, both liquids stained with methylene blue; and (**b**) FT-IR spectra of LLB-PF and LLB-CF.

The wettability of as-prepared absorbents was a crucial factor in selective oil and organic solvent absorption. The surface hydrophobic and superoleophilic properties of LLB-PF were quantified with a water droplet contact angle and N,N-dimethylformamide stained with methylene blue. Under the cooperation of surface chemical compositions and large/minute-scale bubbles structure, the absorbents possessing hydrophobic and superoleophilic property. While LLB-PF samples completely absorbed N,N-dimethylformamide because of their superoleophilicity, it could also support a spherical water droplet on its surface, proving its remarkable hydrophobicity (inset in Figure 3a). The contact angles (CA) of the liquid droplet on LLB-PF and LLB-CF were measured using water. It is well-known that the pyrolysis temperature has a great influence on the resultant carbon monoliths [42]. The water contact angles (WCA) of foams are in the range of 144.6–82.8°, decreasing with the rising pyrolysis temperature (Figure 3a). The measurements were made in static contact angle mode using the Laplace–Young calculation method. The LLB-PF showed a WCA of CA-144.6°, while LLB-CF900 showed a WCA of CA-105.6°, LLB-CF1000 showed hydrophilicity (Figure 2a). The differences of the wettability between LLB-PF and LLB-CF could be explained by FT-IR spectroscopy analysis (Figure 3d). The FT-IR spectrum for LLB-PF indicated the presence of hydrophobic functional groups, such as C=C and –CH_2_– of lignin at 1648 and 1478 cm^−1^, respectively, Benzene rings skeleton vibration of the phenyl group at 1601 and 1450 cm^−1^, the –OH of free phenol at 1197 cm^−1^, =C–O–C of benzene ring branch at 1052 cm^−1^, the peak at 877 cm^−1^ is benzene ring adjacent substitution. While carbonized product LLB-CF displayed relatively weak hydrophobic peaks at the same wavelength, and the peak intensity decreased as the pyrolysis temperature increased. For LLB-CF1000 sample, no hydrophobic functional groups were observed, corresponding to its hydrophilicity in Figure 3a. Therefore, LLB-PF exhibited superb hydrophobicity, while LLB-CF900 exhibited good hydrophobicity, benefiting from the relatively weak hydrophobic peaks. The hydrophobicity and superoleophilicity of LLB-PF and LLB-CF900 were demonstrated in this wettability study. The hydrophobic open-cells in LLB-PF and LLB-CF not only provided selective absorb capacity for the recycling of oil and organic solvents from water, but also provided sufficient space for the storage of those solvents and capillary action to spontaneously drive the solvents into the cells [43].

### 3.3. Thermostability

**Figure 4:**
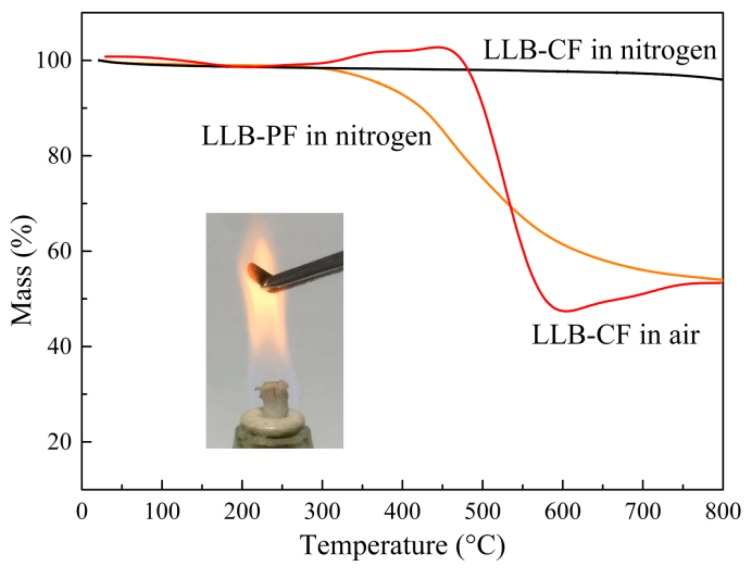
TGA curves for LLB-PF and LLB-CF in a nitrogen atmosphere, LLB-CF in air, respectively. The inset is a photograph of LLB-CF within the flame of an alcohol burner.

The decomposition behaviors of LLB-PF and LLB-CF were observed by thermogravimetric analysis (TGA), as shown in Figure 4. With good thermostability and high carbon content, LLB-PF can act as skeleton to support the 3D interconnected honeycomb porous structure, and finally convert into carbonaceous components of LLB-CF. The TGA curve for LLB-CF in a nitrogen atmosphere was almost constant. Meanwhile, the behavior of LLB-CF in air showed there was only a 5.5-wt % weight loss at 500 °C, mainly resulting from the removal of water absorbed on the cell structure and the destruction of labile hydrophobic chemical components [44]. Therefore, when LLB-CF was exposed to the flame of an alcohol burner, it can resist combustion and remain inert, suggesting superior fire-resistant properties. This property enabled LLB-CF to show recycle through combustion and rendered it reusable for absorbing flammable and useless organics.

### 3.4. Absorption Analysis

**Figure 5:**
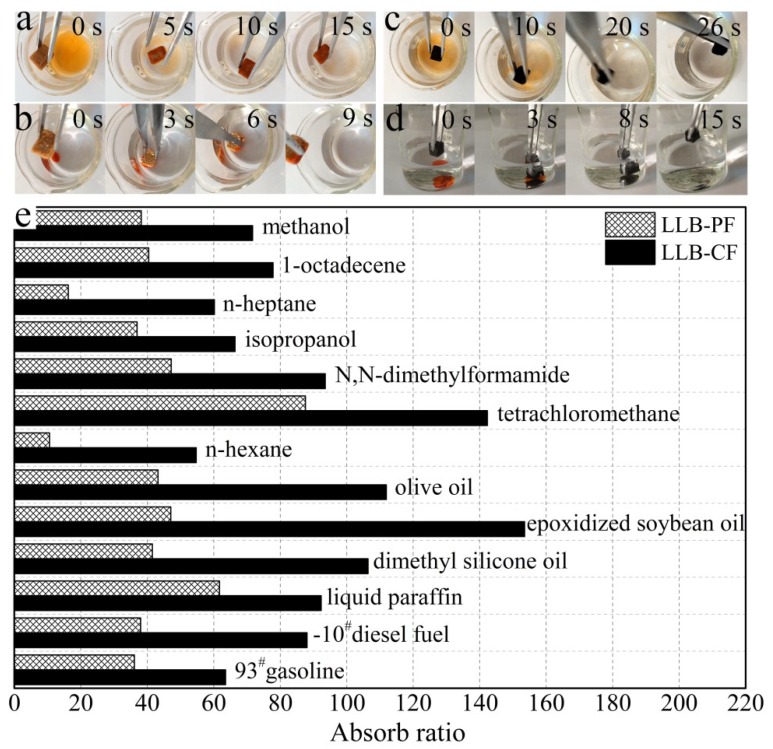
Absorption tests for LLB-PF and LLB-CF. (**a**,**c**) Photographs showing the absorption process of n-heptane using LLB-PF and LLB-CF taken at 15 and 26 s, respectively. N-heptane stained with Sudan red 3 floating on water was completely absorbed; (**b**,**d**) Photographs showing the absorption process of tetrachloromethane using LLB-PF and LLB-CF taken at 9 and 15 s, respectively. Tetrachloromethane stained with Sudan red 3 at the bottom of water was completely absorbed; (**e**) Absorption efficiency of LLB-PF and LLB-CF for oil and organic solvents. The absorption ratio here is defined as the ratio of the absorbate weight to fresh, dried LLB-PF and LLB-CF self-weights.

The remarkable absorption capabilities of LLB-PF and LLB-CF are shown in Figure 5. When LLB-PF and LLB-CF were contacted with an n-heptane layer stained with Sudan red 3 on a water surface, they absorbed the n-heptane completely and rapidly within 15 and 26 s, respectively (Figure 5a,c). Because of their low density and hydrophobicity, both absorbents floated on the water surface after absorbing n-heptane, indicating their potential use for the facile recycling of oil spills and chemical leaks and ease of recycling. In addition, they could also be used to absorb tetrachloromethane quickly, which was stained with Sudan red 3 at the bottom of the water layer (Figure 5b,d).

To study the absorption capacity quantitatively, the absorption ratio was defined as the weight of absorbed substance per unit of weight of the fresh dried LLB-PF and LLB-CF. Various types of oil and organic solvent were studied, such as commercial petroleum products (e.g., 93^#^ gasoline and −10^#^ diesel fuel), seed oils (e.g., epoxidized soybean oil and olive oil), alkanes with different carbon chain lengths, methanol, isopropanol and n-heptane. These materials are common pollutants encountered in our daily life and in industry. Both LLB-PF and LLB-CF showed a very high absorptive capacities for all of the above oil and organic solvents. The absorption ratio for LLB-CF was much higher than for LLB-PF, because of the lower density of LLB-CF. In general, LLB-PF can take up these solvents at 11 to 88 times its own weight, with a maximum of 88 times (for tetrachloromethane). LLB-CF can take up these solvents at 55–153 (epoxidized soybean oil) times its own weight (Figure 5e).

Importantly, the LLB-PF and LLB-CF prepared from waste sawdust showed a much higher absorption capacity than many foam sorbents prepared from various non-renewable precursors reported in previous research (Table 1) [18,35,43,44,45,46,47,48,49,50], for instance, graphene precursors including a reduced graphene oxide foam (10–37 times) [50], spongy graphene (20–86 times) [47], and magnetic graphene foam (10–27 times) [44]; polymer precursors, including modified polyurethane foam (20–32 times) [49]; and coal liquefaction residue precursors, including CN/CF composites (18–28 times) [43]. Although the absorption capacities of LLB-PF and LLB-CF were still lower than that of graphene foam (40–196 times) [18] and melamine sponge/resol based CF (61–203 times) [45], the preparation method for LLB-PF and LLB-CF is the simplest, and the raw material, waste larch sawdust, was cheap. Therefore, with comprehensive consideration of the operation and cost, LLB-PF and LLB-CF are promising absorbents for oil and organic spill recycling.

### 3.5. Recyclability Tests

**Figure 6:**
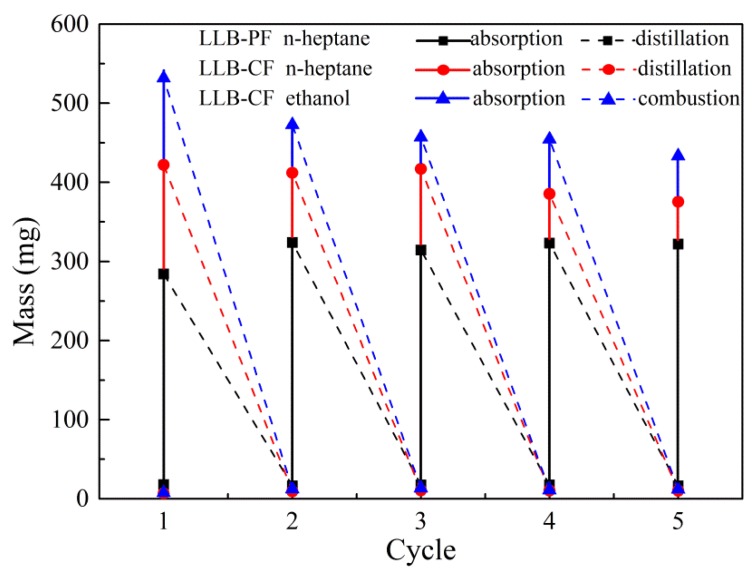
Recyclability of LLB-PF and LLB-CF.

The regeneration of LLB-PF and LLB-CF is illustrated in Figure 6. The recyclability of LLB-PF and LLB-CF and the recoverability of oils and organic solvents are key criteria, because most oils and organics are either useful, valuable raw materials or organic. Repeated recycle tests were performed on LLB-PF and LLB-CF through distillation and combustion. Distillation was used as the main method to recycle valuable pollutants or those with low boiling points. After absorption for n-heptane, foams were heated to the solvent boiling point to release absorbed n-heptane. No obvious change in absorption capacity and morphology was found after five absorption/distillation cycles, because the size and porous structure remained constant during the whole process. Furthermore, direct combustion in air was used as an alternative method for flammable and useless oils or organics, and in this case absorbed ethanol was used efficiently for heating. After five absorption/combustion cycles, there was still no obvious changes for absorption capacity. The results clearly confirmed the good recyclability of LLB-PF and LLB-CF when used as absorbents.

### 3.6. Separation Tests

**Figure 7:**
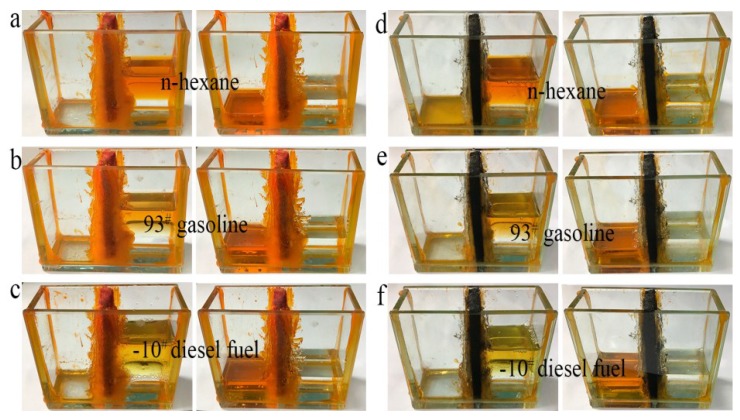
Water/organic solvent mixture separation by LLB-PF and LLB-CF.

An experiment on direct separation of an organic solvent/water mixture was performed using the simple separation device shown in Figure 7. Different from distillation and combustion, direct separation of an organic solvent from water, solely by gravity-driven settling, is the most economical and easiest method, because both distillation and combustion consume manpower and material resources. More importantly, direct separation is more suitable for separating large amounts of solvents, and causes no damage to oil or organics during separation. As shown in Figure 7c,f, water and cyclohexane were separated completely by foam filters (cyclohexane stained with Sudan red 3). When a mixture of water and cyclohexane was poured into one side of the trough, the cyclohexane permeated through the foam filter and passed through to the other side of the trough within 30 min, while all the water remained on the original side. This separation test indicates that as-prepared LLB-PF and LLB-CF filters can separate water and organic solvents effectively.

## 4. Conclusions

In summary, two kinds of form materials, LLB-PF and LLB-CF, with honeycomb-like interconnected open cell structure were prepared from waste larch sawdust. Both form materials show remarkable and fast absorption for oil and/or organic solvents due to their unique open cell structure with abundant pores, hydrophobicity, and superoleophilicity. LLB-PF and LLB-CF could absorb up to 88 and 153 times for oils and/or organic solvents, respectively. LLB-PF and LLB-CF also showed excellent absorption capacities even after five cycles. The excellent oil/organic solvent absorption capability along with the renewable and low-cost waste wood-based materials make our developed LLB-PF and LLB-CF form materials promising for practical water cleanup of the oil spills and organic solvents.

## Figures and Tables

**Table 1:** Comparison of various foam absorbents.

Precursors	Absorbents	Absorption (g/g)	Cost	Ref.
graphene oxide suspension	reduced graphene oxide foam	10–37	high	[49]
graphene oxide film	magnetic graphene foam	12–27	medium	[43]
oxidizing expandable graphite	spongy graphene foam	20–86	high	[46]
coal liquefaction residue	CN/CF composite	18–28	high	[42]
polyurethane foam	modified polyurethane foam	20–32	medium	[48]
graphite flakes	graphene foam	40–196	high	[18]
sponge and resol	CF	61–203	low	[44]
alkaline lignin and melamine	carbon aerogels	5–12	medium	[45]
synthesized SiO_2_ monolith	CF	23–48	high	[47]
lignin	lignin-based polyurethane/graphene oxide foam	26–68	high	[35]
larch sawdust	LLB-PF	11–88	quite low	This work
larch sawdust	LLB-CF	55–153	quite low	This work
